# Bond Properties Between Bimetallic Steel Bar and Polyoxymethylene Fiber-Reinforced Seawater Sea–Sand Concrete

**DOI:** 10.3390/polym17212866

**Published:** 2025-10-27

**Authors:** Fei Wang, Xuanyi Xue, Neng Wang, Shuai Li, Zhengtao Yang, Yuruo Chang

**Affiliations:** 1 School of Civil Engineering, Chongqing University, Chongqing 400045, China; 27130461@alu.cqu.edu.cn (F.W.); xuexuanyi@cqu.edu.cn (X.X.); 20221601050@stu.cqu.edu.cn (Z.Y.); 2 State Key Laboratory of Safety and Resilience of Civil Engineering in Mountain Area, Chongqing 400045, China; 3 School of Management Science and Real Estate, Chongqing University, Chongqing 400045, China; 202403041453@stu.cqu.edu.cn; 4 Department of Civil Engineering, The University of Hong Kong, Pokfulam Road, Hong Kong 999077, China; ls-shuai.li@hku.hk

**Keywords:** polyoxymethylene fiber, fiber-reinforced concrete, bond properties, bimetallic steel bar, constitutive model

## Abstract

With the development of infrastructure construction, seawater sea–sand concrete (SWSSC) is expected to solve the shortage of freshwater and river sand. Polyoxymethylene (POM) fiber, owing to its excellent corrosion resistance, provides a novel approach to enhancing the bond performance of SWSSC. This study systematic study of the bond properties of bimetallic steel bars (BSBs) in POM fiber-reinforced SWSSC and develops a predictive model. Mechanical property tests of SWSSC and pull-out tests of BSB and SWSSC were conducted with various POM fiber contents. The results showed that the optimal volume fraction of POM fibers was 0.6%. At this fraction, the compressive strength and splitting tensile strength of SWSSC were improved by 17.7% and 20.3%, respectively, compared with the group without fibers. All pull-out specimens experienced splitting failure. The bond strength increased monotonically with the increase in relative cover thickness and exhibited a trend of first increasing and then stabilizing with rising POM fiber volume fraction. In addition, a bond stress–slip prediction model between BSBs and POM fiber-reinforced SWSSC was established based on the test results, which can provide theoretical support for the numerical simulation and design of BSB-SWSSC structures.

## 1. Introduction

Fiber-reinforced concrete (FRC) has emerged as a promising construction material in civil engineering, owing to its enhanced mechanical properties and durability compared to conventional concrete [1,2,3]. As a critical category of reinforcing fibers, polymer fibers have gained significant attention for their ability to mitigate cracking, improve tensile strength, and enhance the toughness of concrete matrices [4,5,6]. Polymer fibers effectively bridge microcracks, restrain crack propagation, and contribute to better energy absorption, making them suitable for various structural applications, including high-performance buildings, marine structures, and infrastructure under harsh environments [7]. Compared with other polymer fibers, the polyoxymethylene (POM) fiber exhibits excellent alkali and corrosion resistance, essential for long-term performance in aggressive environments [8,9]. Additionally, POM fibers feature a moderate tensile strength and elongation, along with a smooth monofilament structure that minimizes adverse effects on concrete workability by reducing agglomeration [10]. These characteristics make POM fibers an advantageous material for reinforcing concrete in corrosive conditions.

The increasing demand for infrastructure development has exacerbated the scarcity of traditional construction materials, such as freshwater and river sand [11,12,13]. Seawater sea–sand concrete (SWSSC) has emerged as a sustainable solution to address this challenge by utilizing abundant seawater and sea–sand as raw materials, enabling in situ resource utilization and reducing reliance on freshwater and river sand [14,15,16,17]. Wang et al. [18] and Wang et al. [19] also believed that the SWSSC can be more eco-friendly by incorporating a combination of limestone, calcined clay and cement ternary system. However, SWSSC contains high concentrations of chloride ions and sulfate ions, which may reduce the durability of the structure [20,21]. Therefore, it is necessary to use SWSSC in combination with durable fibers and reinforcement bars [22,23,24].

The bond behavior between steel bars and concrete is a key factor in stress transfer between steel bars and concrete, which ensures the bearing capacity of structures [25]. In recent years, many researchers have studied the bond performance of FRC, including the influence of fiber type, fiber content, and concrete strength. Chu et al. [26] studied the bond performance of the steel bar and steel fiber-reinforced concrete, and proposed a bond stress–slip model for reinforced concrete that considers the influence of steel fibers. Sagar et al. [27] investigated the effect of silica fume and glass fiber on the bond performance of concrete. The experimental results showed that when containing 5% silica fume and 0.25% glass fiber, the concrete specimens exhibited better bond performance. Farhan et al. [28] found that steel fibers can notably enhance the bond force between geopolymer concrete and steel bar. Bediwy and El-Salakawy [29] proposed an empirical model to estimate the bond strength of FRC specimens based on 413 pull-out specimens. Zhou et al. [30] conducted an experimental study on the bond performance of POM fiber-reinforced concrete, and discussed the effect of the compressive strength of concrete. The bond stress–slip model is an important representation of bonding behavior. Liang et al. [31] found that the traditional bond stress–slip model of steel bar and concrete was not applicable to fiber-reinforced bars and concrete. Liu et al. [32] employed a continuous-damage bond–slip model in the study on polypropylene fiber-reinforced concrete. The model introduced bond stiffness to describe the bond–slip relationship between reinforcement and concrete. Because the bond stiffness was obtained through empirical regression, it might not be directly applicable to other fiber-reinforced concrete studies. In recent years, a variety of advanced composite materials have been discovered and investigated. Shruti et al. [33] reported that jute-cowpea composites exhibit good tensile performance and biodegradability, showing application potential in lightweight, low-load scenarios such as packaging materials and non-structural components. Ravi et al. [34] demonstrated that natural-fiber (hemp and banana) fabric-reinforced polymer composites possess excellent moisture-resistance, markedly superior to unmodified natural-fiber composites. However, as the core material of load-bearing structures, concrete needs to be reinforced with fibers to improve its mechanical properties (especially compressive and tensile strength). The POM fibers selected in this study have a tensile strength of up to 970 MPa and a modulus of elasticity of 8 GPa, combining high strength with moderate ductility. These high-performance characteristics enable POM fibers to effectively enhance the performance of the concrete.

However, studies on bond performance in fiber-reinforced SWSSC remain limited, especially regarding the synergistic effects of polymer fibers and corrosion-resistant rebars. As the mixing water of SWSSC, seawater contains high concentrations of chloride (Cl^−^) and sulfate (SO_4_^2−^) ions, which may affect the mechanical properties of SWSSC. Choi et al. [35] identified that seawater exposure could generate positive effects. During short-term exposure, these effects were manifested in the improvements of flexural strength and durability. Park et al. [36] found that seawater-cured specimens achieved compressive strengths 14.82% and 12.14% higher than those cured in tap water at 7 and 28 days, respectively. Xue et al. [10] also believed that compared to ordinary concrete, seawater concrete has a faster increase in strength at an early age. Therefore, the bonding performance of SWSSC may differ from ordinary concrete due to changes in concrete properties. To address this issue, this study focuses on the bond properties between bimetallic steel bars (BSBs) and POM fiber-reinforced SWSSC. The structure of the rest of this paper is arranged as follows. Section 2 details the materials used in the study, including POM fibers, BSBs, and the mix design of SWSSC. Section 3 describes the experimental program, encompassing specimen preparation, mechanical property tests, and pull-out test procedures. Section 4 presents and discusses the experimental results, including mechanical properties of SWSSC, bond failure modes, bond stress–slip curves, and the effects of fiber content and *c*/*d* on bond parameters. Section 5 proposes a bond–slip constitutive model and validates its accuracy against experimental data.

## 2. Materials

### 2.1. Polyoxymethylene Fiber

**Figure 1:**
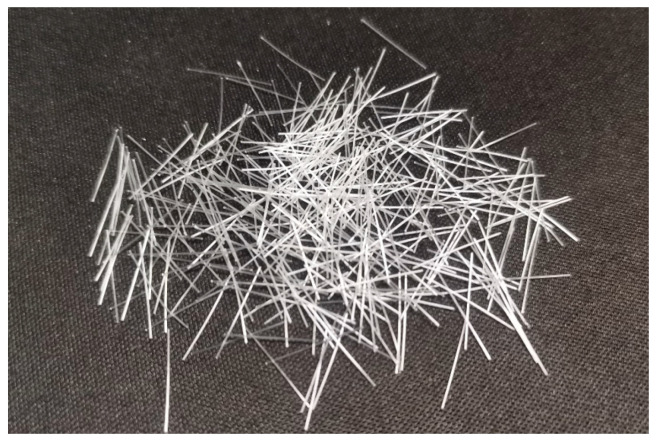
POM fiber [9].

The physical properties of POM fibers used in this study are presented in Table 1. The diameter and length of the POM fiber were 0.2 and 12 mm, respectively (Figure 1). The POM fibers have excellent mechanical properties, with a tensile strength of up to 970 MPa and an elongation at break of 18%. The high tensile strength can improve the properties of concrete. At the same time, its chemical corrosion resistance also makes it durable in SWSSC. Therefore, the POM fiber was selected as an ideal reinforcement material to improve the bond performance between BSBs and SWSSC. It is worth noting that the POM fibers used in this study were in raw monofilament form without any surface treatment or modification. POM fibers inherently possess excellent chemical stability, raw POM fibers can form a stable interface with the SWSSC matrix, and surface modification would not significantly enhance interface bonding but would increase production complexity and costs. Moreover, preserving the raw form ensures SWSSC workability. The smooth surface of raw POM monofilaments minimizes friction between fibers and aggregates, reducing agglomeration risk.

### 2.2. Bimetallic Steel Bar

**Figure 2:**
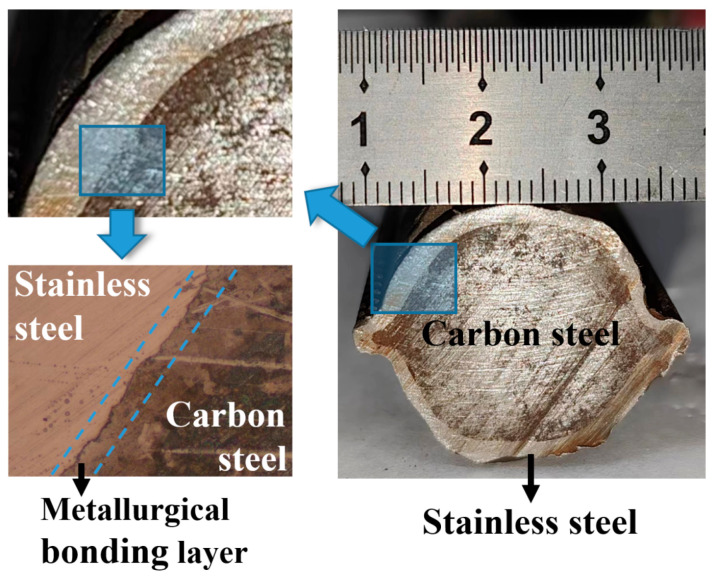
Details of BSBs.

The BSB used in this study consists of an S30408 stainless steel cladding and an HRB400 carbon steel core (Figure 2). Compared with traditional carbon steel reinforcement, this bimetallic structure significantly enhances corrosion resistance of steel bar. The nominal diameter of the reinforcement is 25 mm, and the average thickness of the cladding layer is 2.3 mm. Local cladding thickness measurement results showed that the thickness varied from a minimum of 1.7 mm to a maximum of 3.1 mm. The BSB used in this study adopted a metallurgical bonding process between the S30408 stainless steel cladding and HRB400 carbon steel core, which achieved a metallurgical-grade interface combination (Figure 2). As validated by previous studies [24,37], this bonding form ensured that the cladding and core remained tightly integrated without separation or cracking even under large plastic deformation. After pull-out tests, the cladding of BSB maintained its original integrity. There was no peeling, cracking, or localized deformation of the cladding observed, indicating that the cladding effectively transferred stress with the core during the bond failure process. Therefore, it could be considered that the slight variation in BSB cladding thickness had a negligible impact on localized bonding. The mechanical properties of the BSB are presented in Table 2. The BSB exhibits a tensile strength of 648.4 MPa, and an elongation of 31.3%, thus combining high strength with good ductility. From the microstructure shown in Figure 2, the cladding layer and the core were tightly connected through the metallurgical bonding layer (blue dotted line marked area), ensuring load transfer efficiency between the cladding and core materials during service.

### 2.3. Seawater and Sea–Sand Concrete

Natural seawater and sea sand were used as primary raw materials to prepare SWSSC. The seawater used in the study was sourced from Quanzhou, Fujian Province, China (118.36° E, 24.56° N). Its chemical composition, as shown in Table 3, contained significant amounts of Cl^−^, SO_4_^2−^, and Na^+^ ions, which may pose corrosion risks to concrete structures. The sea sand was sourced from Guangzhou, Guangdong Province, China (113.17° E, 23.79° N). The chloride concentration of the seawater used in this study was 19,365.5 mg/L, which was highly consistent with the typical range of ion concentrations in surface Atlantic seawater [38]. Moreover, the sea sand from Guangzhou employed in this research met the particle-size distribution requirements for ordinary concrete sand stipulated in the Chinese standard JGJ 52-2025 [39]. Therefore, although the seawater and sea sand were taken from a specific location, their compositional characteristics were broadly representative and did not limit the general applicability of the research results. In addition, seawater ions vary slightly with seasons, but the impact of these changes on concrete performance can be ignored. The mix proportions of the POM fiber-reinforced SWSSC are shown in Table 4. The designed strength was C50, with fiber volume fraction (*ρ*) from 0 to 1.0%, to evaluate the effects of different POM fiber contents on the performance of the concrete. The specimens were cured in a concrete standard curing environment with a constant temperature of (20 ± 2) °C and a relative humidity of not less than 95% for a period of 28 days to ensure consistent hydration conditions for all specimens. The standard curing condition meets the requirements of GB/T 50081-2019 and has also been adopted in many studies [10,40,41,42,43]. Curing conditions affect concrete hydration, which in turn impacts bond performance. Unifying to standard curing can eliminate external variable interference, ensuring observed bond property differences are attributed to intrinsic factors (e.g., fiber content, concrete mix proportion), which also facilitates comparison with other studies.

## 3. Experimental Program

### 3.1. Test Specimen

To explore the bond performance between BSB and POM fiber-reinforced SWSSC, mechanical property tests and pull-out tests were carried out. The mechanical property tests included compressive strength tests and splitting tensile strength tests. The specimens were designed as 150 × 150 × 150 mm cubes, incorporating six different *ρ* (0%, 0.2%, 0.4%, 0.6%, 0.8%, and 1.0%). Three duplicate specimens were included in each variable group.

**Figure 3:**
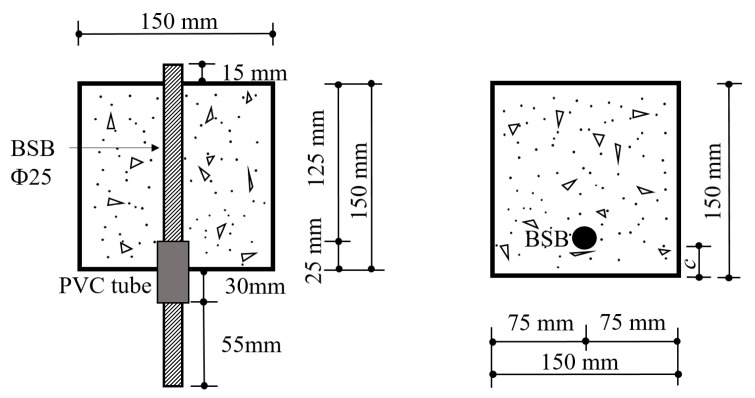
Dimensions of pull-out specimens.

The design of the pull-out test specimens is illustrated in Figure 3. The length of the composite reinforcement was 250 mm, and the bond length (*Lₑ*) was five times the diameter of the BSB. An unbonded zone of 25 mm was set at the loading end to reduce the influence of concrete compression on the bond behavior. The unbonded section was achieved by isolating the BSB from the concrete using a PVC pipe. The pull-out specimens also included the six aforementioned fiber contents, and an additional variable of three different ratios of concrete cover thickness to reinforcement diameter (*d*) (*c*/*d* = 1, 1.8, and 2.6) was considered, as presented in Table 5, where *c* is the concrete cover thickness, and *d* is the reinforcement diameter. A total of 54 pull-out specimens were prepared.

### 3.2. Mechanical Property Test

**Figure 4:**
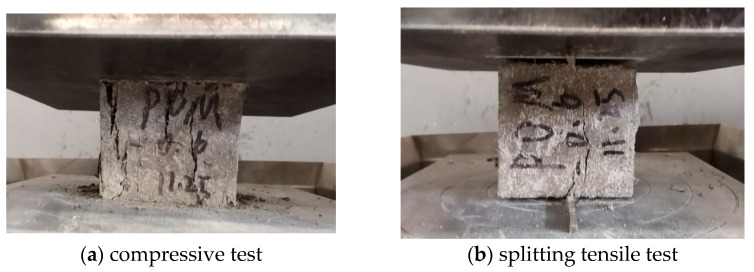
Mechanical property tests of SWSSC.

The mechanical property tests of POM fiber-reinforced SWSSC were carried out in accordance with the GB/T 50081-2019 [44]. These tests aimed to determine the mechanical properties of SWSSC with different POM fiber contents, providing basic parameters for bond behavior research. A SHT-Y3000 electro-hydraulic servo pressure testing machine (New Ssans Enterprise Development Co., Ltd, Shanghai, China) was used to conduct the compressive strength tests and splitting tensile strength tests (Figure 4). In the compressive strength tests, the loading rate was controlled at 0.5 MPa/s to ensure that the load was uniformly applied. A loading rate of 0.05 MPa/s was adopted for the splitting tensile strength tests, and linear loads were applied via steel cushion strips to induce the specimens to split.

### 3.3. Pull-Out Test

**Figure 5:**
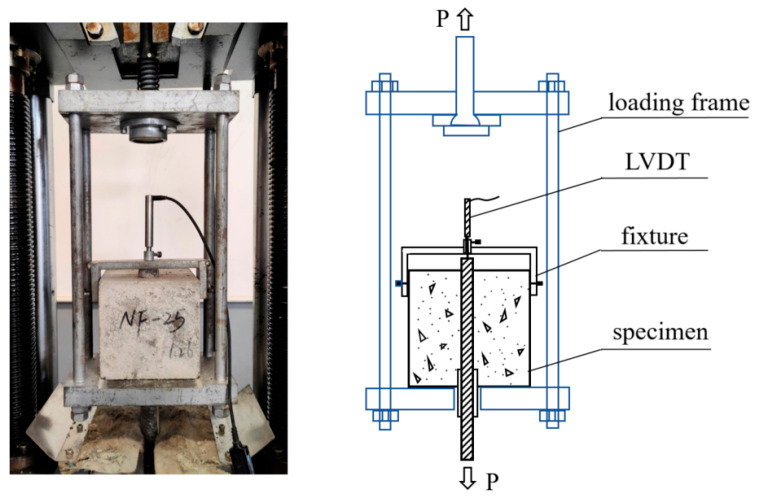
Pull-out test system.

Pull-out tests were conducted on an SHT-600 servo-controlled testing machine (New Ssans Enterprise Development Co., Ltd., Shanghai, China), as shown in Figure 5. During the pull-out test, a reaction frame was first positioned in the testing machine, after which the specimens were placed in the reaction frame. An extensometer was fixed at the free end using clamps to record the slip of BSB (*s*_0_). Since the relative slip between the BSB and SWSSC also includes the elongation of the BSB within the bond section, the actual relative slip (*s*) between the reinforcement and concrete was obtained using Equation (1). Pull-out loading (*P*) was carried out at a rate of 0.5 mm/min, and this process continued until the specimens failed. The bond stress of each specimen was computed using Equation (2).

(1) $$s=s_{0}-\frac{F}{E_{S}A_{S}}L_{e}$$

(2) $$\tau =\frac{F}{\pi dL_{e}}$$

where *E_S_* is the elastic modulus of the BSB; *A_S_* is the cross-sectional area of the BSB; and *d* is the diameter of the BSB.

## 4. Results and Discussion

### 4.1. Mechanical Properties

**Figure 6:**
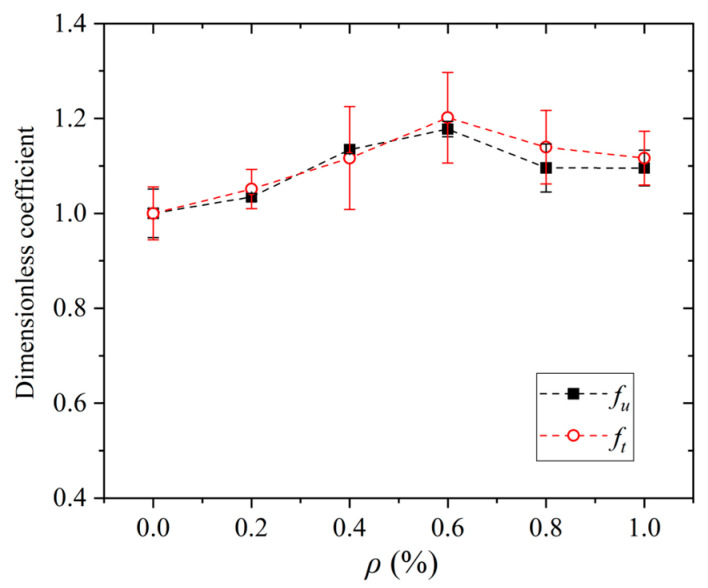
Mechanical performance of SWSSC.

**Figure 7:**
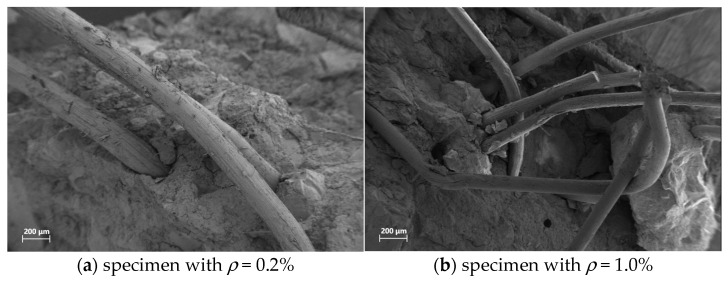
SEM images of POM fiber-reinforced SWSSC.

The mechanical performance of SWSSC is summarized in Table 6 and Figure 6. For the compressive strength (*f_u_*), the control group (NF, *ρ* = 0%) exhibited a strength of 56.4 MPa. As the POM fiber content increased, *f_u_* first increased, reaching a peak of 66.4 MPa at a fiber volume fraction of 0.6%, and then decreased to 61.8 MPa for both 0.8% and 1.0% fiber contents. A similar trend was observed for the splitting tensile strength (*f_t_*). The NF group had a *f_t_* of 3.06 MPa, which rose to a maximum of 3.68 MPa at 0.6% fiber content, before declining to 3.49 MPa (*ρ* = 0.8%) and 3.42 MPa (*ρ* = 1.0%). Figure 7 shows the microstructure of SWSSC with different fiber content. The initial strength increase in mechanical strength with POM fiber addition can be attributed to the fiber’s crack-bridging effect. POM fibers effectively restrain microcrack propagation and enhance load transfer within the concrete matrix. However, excessive fiber content may induce fiber agglomeration, reducing the uniformity of the matrix and impairing workability during casting, thereby deteriorating mechanical performance. Thus, a POM fiber volume fraction of approximately 0.6% optimizes the mechanical properties of SWSSC, balancing fiber reinforcement and matrix homogeneity.

### 4.2. Failure Mode

**Figure 8:**
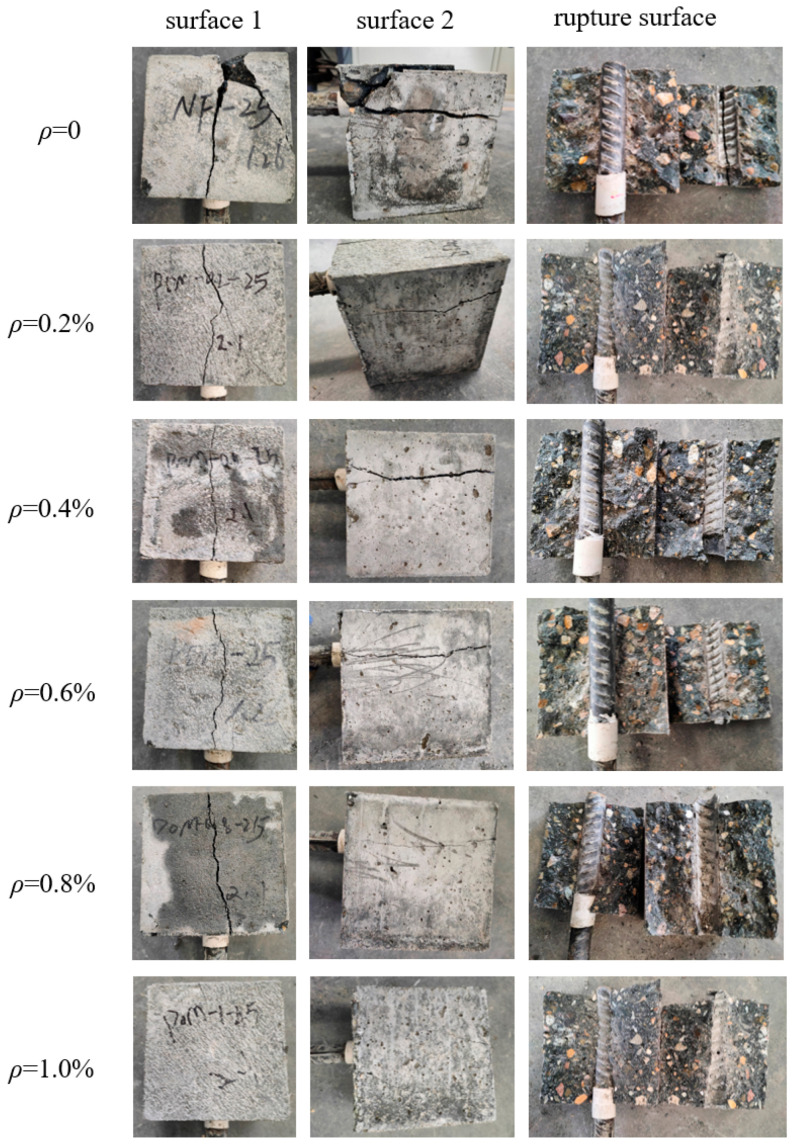
Failure behaviors of specimens.

The bond force between BSBs and SWSSC is composed of friction force, adhesion force, and mechanical interlocking force. After the pull-out tests, all specimens exhibited a splitting failure mode (Figure 8). In the initial loading stage, the slip between the BSB and SWSSC was minimal, as the bond stress was dominated by chemical adhesion. As the loading increased, the slip gradually increased due to the degradation of adhesion force. When the radial stress exceeded the *f_t_* of SWSSC, radial cracks initiated and propagated. Once the load reached its peak, the SWSSC cracked radially from the center of the BSB to the specimen surface, resulting in specimen failure. Specimens without POM fibers showed more severe damage, larger cracks, and were fractured into several large concrete fragments. Although specimens with POM fibers exhibited noticeable cracks on the side surfaces, the cracks were more minor compared to NF specimens. This was because the bridging effect of POM fibers enhanced the constraint of SWSSC on BSB, while improving the integrity of specimens.

### 4.3. Bond Stress–Slip Curve

**Figure 9:**
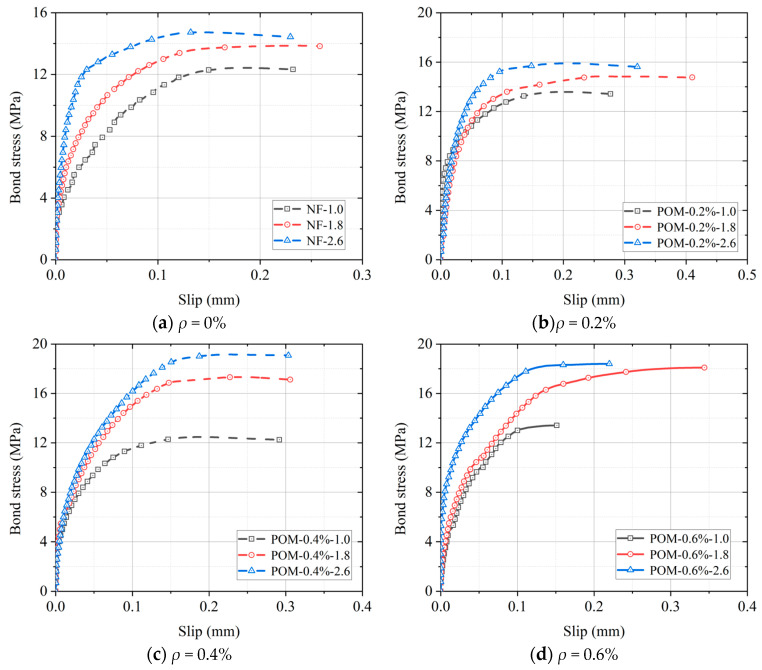

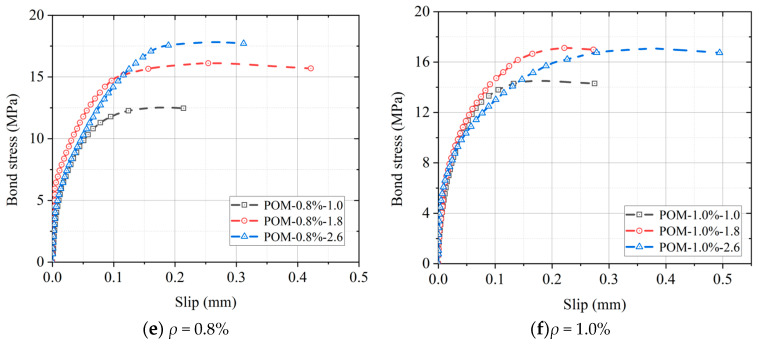
Bond stress–slip curves of specimens.

Based on pull-out tests, the bond behavior between BSB and SWSSC with different POM fiber contents was investigated, as shown in Figure 9. It was observed that the bond stress–slip curves between BSB and SWSSC could be divided into three stages. At the initial loading stage, the bond stress and slip exhibited a linear relationship. During this phase, the bond force was mainly provided by the chemical bond strength between BSB and SWSSC, and the energy absorbed is mainly elastic. The curve displayed linearity, and no significant deformation or cracks were observed on the specimen surface. As the load increased continuously, cracks initiated sequentially from the loading end toward the free end. At this point, the bond stress was primarily provided by mechanical interlocking and friction. The compressive stress in the front-rib region increased, and microcracks gradually developed inside the specimens, forming a crushed zone ahead of the ribs. The radial pressure exerted by the ribs generated circumferential tensile stress in the concrete. This stage showed a marked increase in energy dissipation due to crack propagation, frictional sliding, and micro-structural damage. After reaching the peak bond stress, the slip between the BSB and SWSSC increased, while the bond stress remained approximately constant. When the circumferential stress exceeded the *f_t_* of the SWSSC, radial cracks developed rapidly, leading to splitting failure and rapid loss of bond force. At this stage, the energy dissipation shifted to fracture energy.

**Figure 10:**
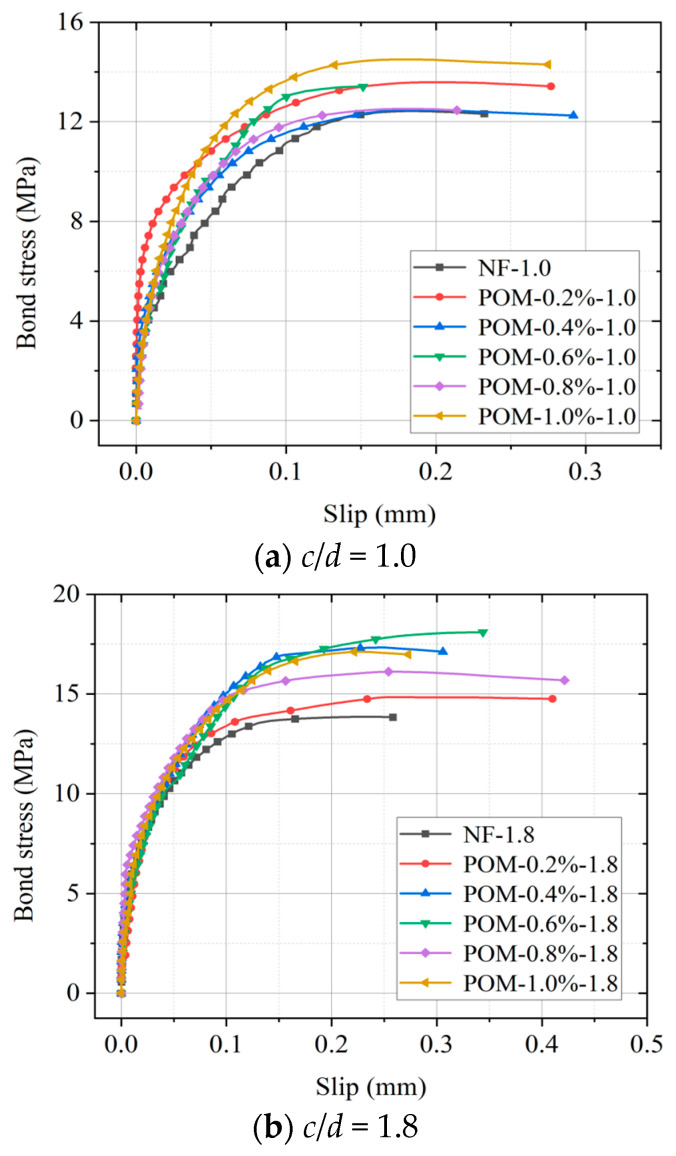

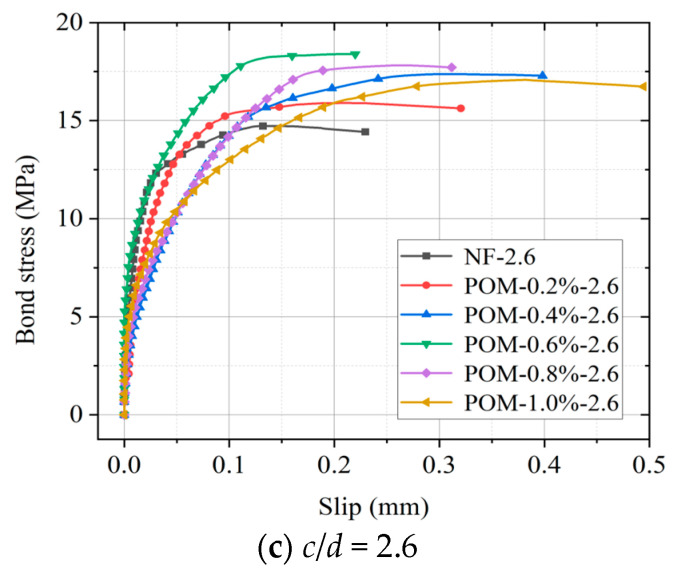
Bond stress–slip curves of specimen.

For specimens with and without POM fibers, the peak bond stress increased correspondingly with the rise in relative cover thickness (*c*/*d*). However, the corresponding slip at the peak was similar. Additionally, as the *c*/*d* increased, the curve became steeper, indicating a faster increase in bond stress with increasing *c*/*d*. It can be attributed to the higher circumferential constraint introduced by the thicker concrete cover. Bond stress–slip curves of SWSSC specimens with various *ρ* are presented in Figure 10. It was found that the specimen with POM fibers exhibited higher peak bond stress, steeper ascending segments of the curve, and longer flat segments after reaching the peak stress. The fiber network adds extra frictional damping and mechanical locking, substantially enhancing the overall energy-absorption capacity, thereby improving the bond toughness.

### 4.4. Bond Properties

**Figure 11:**
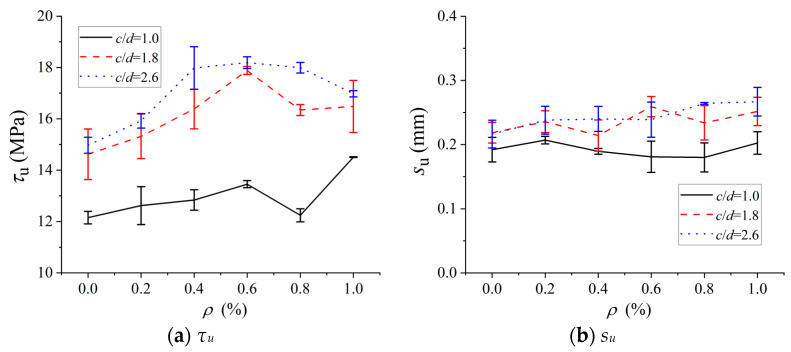
Effects of *ρ* on key parameters of bond stress–slip model: (**a**) *τ_u_*; (**b**) *s_u_*.

**Figure 12:**
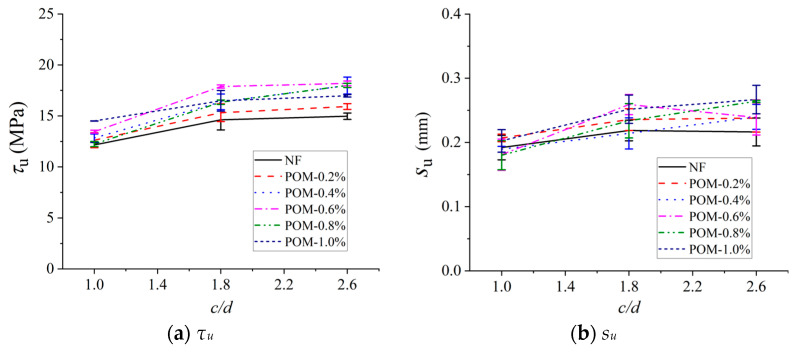
Effects of *c*/*d* on key parameters of bond stress–slip model: (**a**) *τ_u_*; (**b**) *s_u_*.

To investigate the regulatory mechanisms of the POM fiber content (*ρ*) and the *c*/*d* on the bond performance between BSB and POM fiber-reinforced SWSSC, the peak bond stress (*τ_u_*) and the corresponding slip (*s_u_*) were extracted. As shown in Figure 11, the *τ_u_* increased with the increase in *ρ*. The maximum increase in *τ_u_* was observed at a POM fiber content of 0.6%. Based on the mechanical performance test results of SWSSC in Section 4.1, the compressive strength and splitting tensile strength reached their peaks at *ρ* = 0.6%. This indicates that the strength of SWSSC and the interfacial bond performance exhibited similar trends, further validating that 0.6% is the optimal fiber content for POM. It is noteworthy that when the fiber content exceeded 0.6%, the increase in *τ_u_* was significantly reduced. This phenomenon is related to the tendency of fiber agglomeration under excessive POM fiber content. The fiber agglomerates not only destroyed the uniformity of the concrete matrix but also tended to form stress concentration at the interface, which instead weakened the reinforcing effect of fibers on interfacial bonding. In addition, as the *c*/*d* increased, *τ_u_* exhibited a monotonic increase (Figure 12). A thicker concrete cover could effectively resist the radial expansion force exerted by BSBs on the surrounding SWSSC during the pull-out process, delaying the splitting failure of SWSSC and thus enhancing the bond strength. In contrast to the significant variation in *τ_u_*, *s_u_* remained relatively stable, ranging between 0.175 mm and 0.275 mm regardless of the POM fiber content. During the pull-out process, when the BSB began to slide, the transverse ribs of the BSB exerted an oblique pressure on the concrete in front of the ribs. This oblique force induced tearing cracks in the concrete, and the circumferential component of this pressure accelerated the development of these cracks. When the *c*/*d* was relatively small, the circumferential component of the pressure split the concrete, and the cracks would quickly penetrate the protective layer, leading to the failure of the BSBSC specimen. However, when the *c*/*d* was relatively large, the time for cracks to develop to the surface of the specimen increased, which delayed specimen failure and led to a significant increase in *s*_u_. The increase in fibers content can bridge cracks to delay specimen failure. However, its effect was not as significant as increasing *c*/*d*. Based on the experimental results, with the increase in *c*/*d*, *s*_u_ exhibited a monotonically increasing trend, indicating that *s*_u_ was more sensitive to *c*/*d*.

**Figure 13:**
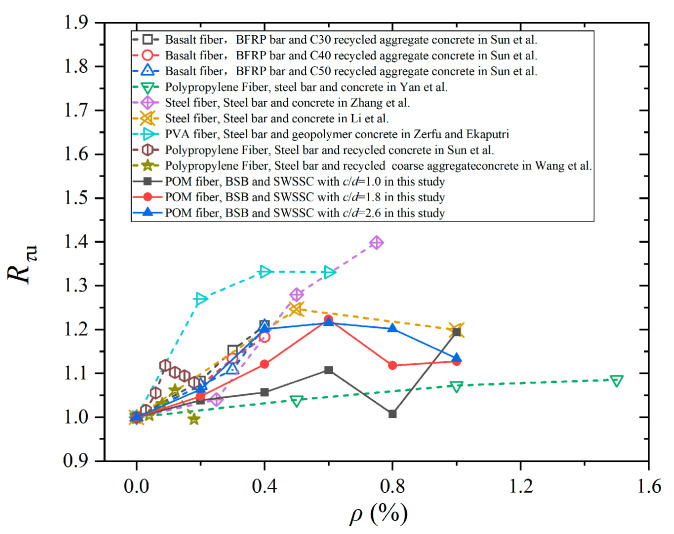
Comparison in bond strength among different fiber-reinforced concretes [45,46,47,48,49,50,51].

Test results for different types of fiber-reinforced concrete (specifically basalt, steel, polypropylene (PP), polyvinyl alcohol (PVA), and POM fiber-reinforced concretes) are compared in Figure 13. The *R_τu_* represents the ratio of the bond strength of fiber-reinforced specimens to that of plain concrete. It can be observed that different fiber types and fiber content exhibited varying enhancement effects on bond strength. As shown in Figure 12, steel fiber-reinforced concrete showed a notable increase in *R_τu_* compared to many other fiber types at similar fiber content. In contrast, the enhancement of PP fibers was the least significant. In addition, PVA fibers exhibited a favorable bond strength enhancement effect at low fiber content. The impact of POM fibers on the bond strength of reinforced concrete was more sensitive to fiber content. When the fiber content was low, POM fibers led to a rapid increase in bond strength. However, when the fiber content exceeded a certain level (0.8%), the enhancement effect on bond performance weakened significantly.

## 5. Bond Stress–Slip Constitutive Model

### 5.1. Bond Stress–Slip Model

**Figure 14:**
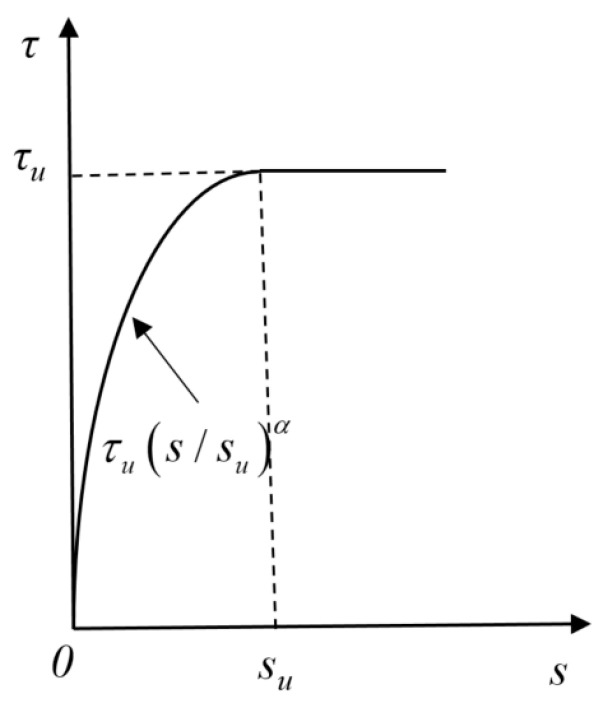
Bond stress–slip model.

To better understand the bond mechanical behavior between POM fiber-reinforced SWSSC and BSBs, the bond stress–slip model framework described by Equation (3) was adopted to characterize the bond stress–slip curves. Equation (3) was adapted from prior studies on reinforcement-concrete interface behavior that have been widely validated for capturing the evolutionary process of bond behavior [43,52,53]. It is important to clarify that while the model framework is adapted from established research, the key model parameters in Equation (3) were derived from regression analysis of our 54-group experimental data, ensuring they specifically reflect the bond characteristics between BSBs and POM fiber-reinforced SWSSC under the tested conditions. As shown in Figure 14, this model describes the relationship between bond stress and slip using a piecewise function, where *s* represents the slip and *τ* represents the bond stress. When the slip value was less than *s_u_*, the nonlinear growth of bond stress with slip was described by an exponential function. The shape parameters of this segment of the bond stress–slip curve were described by the parameter *α*. According to the test results in Section 4.3, the bond–slip curves of specimens did not exhibit an effective descending segment. Therefore, the equation was applicable to both the ascending segment and the plateau segment. When the slip exceeded *s_u_*, the bond stress no longer increased. Based on the experimental results, a mathematical model of the key parameters of the bond stress–slip curve was established considering *ρ* and *c*/*d*, as shown in Equations (4)–(7), where *p*_1_–*p*_9_ are model coefficients. The values of *p*_1_–*p*_9_ were determined using the least squares method, as shown in Table 7 and Table 8. It is worth noting that, the bond stress–slip model developed via least squares regression, derives its parameter predictions from a comprehensive dataset of 54 pull-out specimens in this study. The dataset covers six POM fiber content (*ρ* = 0–1.0%) and three relative cover thicknesses (*c*/*d* = 1, 1.8, 2.6), with 3 replicates per variable combination, ensuring the regression results fully reflect the core relationships between key influencing factors (*ρ*, *c*/*d*) and bond behavior. However, the model may not cover all engineering scenarios. In future research, it is recommended to include more influencing factors and expand the experimental dataset to optimize the model.

(3) $$\tau =\left\{\begin{array}{l} \tau_{u}{\left(s/s_{u}\right)}^{\alpha }\;0\leq s\leq s_{u}\\ \tau_{u}\;s>s_{u} \end{array}\right.$$

(4) $$c/d=1.0\;\;\;\tau_{u}=\left\{\begin{array}{l} p_{1}\rho^{2}+p_{2}\rho +p_{3}\;0\leq \rho \leq 0.8\\ p_{4}\rho +p_{5}\;\;\;\;\;\;0.8<\rho \leq 1.0\; \end{array}\right.$$

(5) $$c/d=1.8,\;2.6\;\;\;\tau_{u}=p_{1}\rho^{2}+p_{2}\rho +p_{3}\;0\leq \rho \leq 1.0$$

(6) $$s_{u}=p_{6}\frac{c}{d}+p_{7}$$

(7) $$\alpha =p_{8}\frac{c}{d}+p_{9}$$

### 5.2. Verification of the Proposed Models

**Figure 15:**
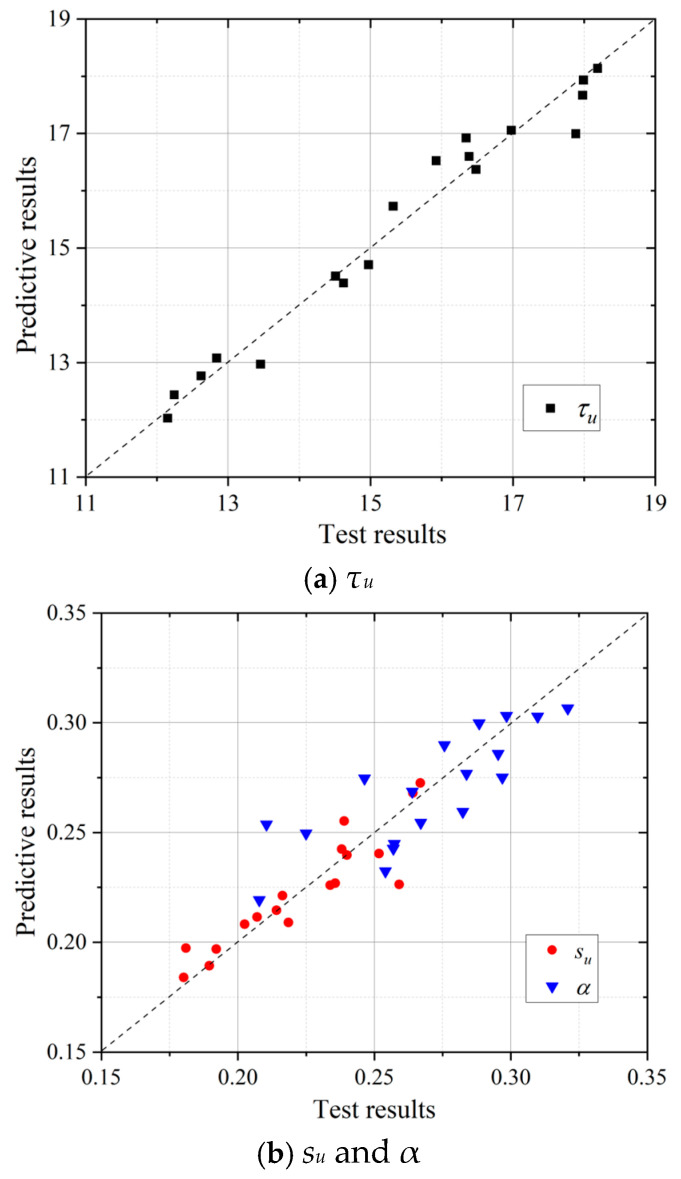
Validation of predictive model: (**a**) *τ_u_*; (**b**) *s_u_* and *α*.

**Figure 16:**
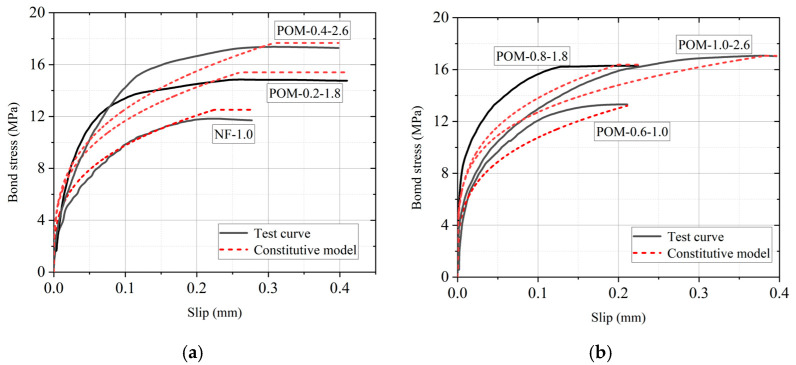
Comparison in bond stress–slip curve between test results and calculated results: (**a**) *ρ* = 0, 0.2, and 0.4; (**b**) *ρ* = 0.6, 0.8, and 1.0.

To evaluate the accuracy of the proposed bond stress–slip model, the calculated values of four key parameters derived from Equations (4)–(7) were compared with the experimental results, as shown in Figure 15. It can be observed that most data points are concentrated around the diagonal line, indicating that the calculated results from the proposed bond stress–slip model were consistent with the experimental results. By substituting the model parameters into Equation (3), the predicted bond–slip curves were obtained, as illustrated in Figure 16. The comparison between the predicted curves and experimental results demonstrated that the proposed bond stress–slip model can adequately describe the bond behavior of BSB and POM reinforced SWSSC. To quantitatively assess the accuracy of the proposed bond stress–slip model, statistical error metrics of *τ_u_* and *s_u_* were calculated, as shown in Table 9. These error metrics confirm that the proposed bond stress–slip model has high accuracy in predicting both *τ_u_* and *s_u_*, and thus can reliably characterize the bond behavior between BSBs and POM fiber-reinforced SWSSC.

## 6. Conclusions

In this study, the effects of POM fiber on the bond behavior between BSB and POM fiber-reinforced SWSSC were investigated. The main conclusions are summarized as follows:

1. The *f_u_* and *f_t_* of SWSSC increased initially and then decreased with increasing POM fiber content, with an optimal fiber volume fraction of 0.6%. At this optimal content, *f_u_* increased from 56.4 MPa to 66.4 MPa, and *f_t_* increased from 3.06 MPa to 3.68 MPa, representing increases of 17.7% and 20.3%, respectively.
2. The bond failure mode of the BSB and POM fiber reinforced SWSSC was splitting failure. Specimens without POM fiber exhibited wider cracks and more severe fragmentation, whereas POM fiber reinforced specimens showed finer cracks and better specimen integrity due to the bridging effect of the fibers. As the *c*/*d* increased, the ascending segment of the bond stress–slip curve became steeper.
3. The *τ_u_* increased with increasing POM fiber content, reaching its maximum at 0.6%. Beyond this content, the growth of *τ_u_* slowed down due to fiber agglomeration. Thicker concrete cover effectively resisted the radial expansion force exerted by BSB on the surrounding concrete during pull-out, thereby delaying the splitting failure of SWSSC. *τ_u_* exhibited a monotonically increasing trend with *c*/*d*. Compared to specimens with *c*/*d* = 1, those with *c*/*d* = 2.6 showed an approximately 35% increase in *τ_u_*. Notably, *s_u_* was significantly more sensitive to the cover thickness than to the fiber content.
4. A predictive mathematical model for key parameters (*τ_u_*, *s_u_*, and *α*) was developed. Based on the bond stress–slip curves obtained from pull-out tests, a piecewise bond stress–slip constitutive model was proposed. By comparing the model-predicted values with the experimental results, it was demonstrated that the proposed model could accurately describe the bond behavior between BSB and POM fiber reinforced SWSSC, providing a theoretical basis for engineering design and numerical simulation.

This study establishes the systematic bond–slip model for BSB and POM fiber-reinforced SWSSC, addressing a critical gap in marine infrastructure applications. Based on 54-group test, the effects of POM fibers on the bond performance of BSB and SWSSC were revealed, which provides a new perspective for improving the bonding durability of SWSSC in marine environments. However, the research on fiber-reinforced SWSSC is still in its infancy. In future research, the following research questions are worth considering: (i) long-term durability assessments of fiber-reinforced SWSSC; (ii) optimization of fiber content; (iii) feasibility of combining low-carbon cementitious materials with seawater and sea sand; and (iv) life-cycle cost analyses of POM-SWSSC systems in marine environments.

## Figures and Tables

**Table 1:** Physical properties of POM fibers.

Material	Tensile Strength (MPa)	Elastic Modulus (GPa)	Elongation (%)	Density (kg/m^3^)
POM fiber	970	8	18	1400

**Table 2:** Mechanical properties of the BSB.

Material	Yield Tensile Strength (MPa)	Ultimate Tensile Strength (MPa)	Elastic Modulus (GPa)	Elongation (%)
BSB	472.5	648.4	191.2	31.3

**Table 3:** Chemical compositions of seawater (mg/L).

Chemical Composition	Cl^−^	SO_4_^2−^	Na^+^	K^+^	Mg^2+^	Ca^2+^
Content	19,365.5	2537.5	11,208.7	389.9	1321.7	395.8

**Table 4:** Mix proportions of SWSSC (kg/m^3^).

Groups	Cement	Fly Ash	Mineral Powder	Sea–Sand	Coarse Aggregate	Seawater	Fiber
NF	264	88	88	831	1016	160	0.0
POM-0.2							2.8
POM-0.4							5.6
POM-0.6							8.4
POM-0.8							11.2
POM-1.0							14.0

**Table 5:** Details of pull-out specimens.

Specimen Number	*c*/*d*	*ρ*	Replicates
NF-*c*/*d*	1, 1.8, 2.6	0	3
POM-*ρ*-*c*/*d*	1, 1.8, 2.6	0.2%, 0.4%, 0.6%, 0.8%, 1.0%	3

Note: NF indicates that POM fibers have not been added.

**Table 6:** Mechanical performance of SWSSC.

Mechanical Properties	NF	POM-0.2	POM-0.4	POM-0.6	POM-0.8	POM-1.0
*f_u_* (MPa)	56.4	58.3	64.0	66.4	61.8	61.8
*f_t_* (MPa)	3.06	3.22	3.42	3.68	3.49	3.42

Note: “NF” refers to SWSSC without POM fibers, while “POM-0.2” to “POM-1.0” denote SWSSC with POM fiber volume fractions of 0.2%, 0.4%, 0.6%, 0.8%, and 1.0%, respectively.

**Table 7:** Key parameters of predictive model for *τ_u_*.

Coefficients	*c*/*d* = 1.0	*c*/*d* = 1.8	*c*/*d* = 2.6
*p*_1_	−5.30	−5.90	−8.41
*p*_2_	4.75	7.88	10.76
*p*_3_	12.03	14.39	14.71
*p*_4_	11.33	—	—
*p*_5_	3.18	—	—

**Table 8:** Key parameters of predictive model for *s_u_* and *α*.

Coefficients	NF	POM-0.2	POM-0.4	POM-0.6	POM-0.8	POM-1.0
*p*_6_	0.01519	0.01936	0.03152	0.03623	0.05252	0.04021
*p*_7_	0.1817	0.1921	0.1578	0.1611	0.1315	0.1680
*p*_8_	−0.03994	−0.01627	0.02158	−0.02673	0.00606	−0.05034
*p*_9_	0.3466	0.3191	0.2471	0.3019	0.2388	0.3501

**Table 9:** Statistical error metrics for model predictions of *τ_u_* and *s_u_*.

Evaluation Index	R^2^	MSE	RMSE	MAE	MAPE
*τ_u_*	0.9685	0.1284	0.3583	0.2776	1.80%
*s_u_*	0.8381	0.0001	0.0109	0.0079	3.46%

## Data Availability

Data is contained within the article.

## Nomenclature

The following abbreviations are used in this manuscript:

FRC	fiber-reinforced concrete
SWSSC	seawater sea–sand concrete
POM	polyoxymethylene
BSB	bimetallic steel bar
*c*/*d*	ratios of concrete cover thickness to reinforcement diameter
*ρ*	fiber volume fraction, %
*s*_0_	slip of the BSB, mm
*s*	relative slip between the BSB and concrete, mm
*E_S_*	elastic modulus of the BSB, MPa
*A_S_*	cross-sectional area of the BSB, mm^2^
*d*	diameter of the BSB, mm
*f_u_*	compressive strength of SWSSC, MPa
*f_t_*	splitting tensile strength of SWSSC, MPa
*τ_u_*	peak bond stress, MPa
*s_u_*	relative slip at peak bond stress, mm
*α*	coefficient of the bond stress–slip model
*p*_1_–*p*_9_	coefficient of the predictive model
